# Curzerene suppresses progression of human glioblastoma through inhibition of glutathione S‐transferase A4

**DOI:** 10.1111/cns.13800

**Published:** 2022-01-20

**Authors:** Bo Cheng, Xiaoliang Hong, Linfang Wang, Yuanyuan Cao, Dengli Qin, Han Zhou, Dianshuai Gao

**Affiliations:** ^1^ Department of Neurobiology and Cell Biology Xuzhou Medical University Xuzhou China; ^2^ Department of Psychiatry The affiliated Xuzhou Oriental Hospital of Xuzhou Medical University Xuzhou China; ^3^ Department of Gynaecology Xuzhou Maternity and Child Health Care Hospital 3 Xuzhou China

**Keywords:** 4‐hydroxynonenal, Curzerene, glioblastoma, glutathione, glutathione S‐transferase A4, mTOR

## Abstract

**Aims:**

Glioblastoma is the central nervous system tumor with the highest mortality rate, and the clinical effectiveness of chemotherapy is low. Curzerene can inhibit the progression of non‐small‐cell lung cancer, but its role in glioma has not been reported. The purpose of this study was to clarify the effect of curzerene on glioma progression and further explore its potential mechanism.

**Methods:**

The expression of glutathione S‐transferase A4 (GSTA4) in glioblastoma and the effect of curzerene on the expression of GSTA4 and matrix metalloproteinase 9 and the activation of the mTOR pathway were detected by Western blotting and RT‐PCR, and the effects of curzerene treatment on glioma malignant character were detected by cell biological assays. The in vivo antitumor effects of curzerene were analyzed in a nude mouse xenograft model.

**Results:**

Curzerene was found to inhibit the expression of GSTA4 mRNA and protein in U251 and U87 glioma cells, and this effect correlated with a downregulation of the proliferation of these cells in a time‐ and dose‐dependent manner. Invasion and migration were also inhibited, and curzerene treatment correlated with induction of apoptosis. Curzerene inhibited the activation of the mTOR pathway and the expression of matrix metalloproteinase 9, and it correlated with increased 4‐hydroxynonenal levels. In vivo, curzerene was found to significantly inhibit tumor growth in nude mice and to prolong the survival time of tumor‐bearing nude mice.

**Conclusion:**

In conclusion, inhibition of GSTA4 correlates with positive outcomes in glioma models, and thus, this molecule is a candidate drug for the treatment of glioma.

## Graphical abstract

Curzerene can suppress glioblastoma both in vivo and in vitro. Curzerene can affect the migration and invasion of glioblastoma by downregulating MMP9 and EMT, and can also inhibit the proliferation and promote apoptosis of glioblastoma by inhibiting the mTOR pathway and downregulating GSTA4

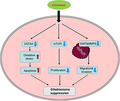

## 1 INTRODUCTION

Glioma is the most common cancer of the human central nervous system, and it is also the most lethal tumor type.^1^ According to data from the International Agency for Research on Cancer (IARC) regarding the global cancer burden, tumors of the central nervous system, including gliomas, caused approximately 30,000 deaths in China in 2020, making glioma the eighth most lethal tumor type in China. Glioma tumors progress rapidly and readily migrate to the surrounding brain tissue, and they are highly insensitive to chemoradiotherapy.^2^, ^3^ The 5‐year survival rate remains less than 5%, even if patients are treated in a timely manner with surgical resection combined with adjuvant chemotherapy.^4^, ^5^

Chemotherapy is an important adjuvant treatment for glioma, but the efficacy of traditional first‐line chemotherapy drugs, including temozolomide, in glioma cases is less than 60%, and their toxicity and side‐effect profiles bring an additional health burden to patients.^6^ Because of unsatisfactory results with front‐line agents, some researchers have begun to explore the inhibitory effects of traditional Chinese medicines on tumors. Some traditional Chinese medicines have been proven not only to lead to lower decreases in patient quality of life, but also to have considerable potent anticancer effects.^7^

*Curcuma longa*, a form of turmeric, is one such traditional Chinese medicine that may represent a source of anticancer agents. Extracts from *Curcuma longa* have been used in the treatment of eye diseases and liver cancer. One compound in these extracts, curzerene, has been shown to downregulate the expression of glutathione S‐transferase (GST) A1 in lung cancer, and it has shown tumor‐inhibiting properties, while exhibiting low toxicity and a lack of damage to the organs of nude mice.^8^ However, while this specific GSTA inhibitor has shown promise for the treatment of cancer, previous research has not explored the impact of curzerene on the occurrence and development of glioma.

Glutathione S‐transferase A4 is an important phase 2 detoxification enzyme that catalyzes redox reactions between glutathione and factors leading to intracellular and extracellular damage, and it also maintains the redox homeostasis of the intracellular environment. Importantly, members of the GSTA family can also affect a variety of cancer‐relevant signaling pathways, thus potentially participating in the occurrence and development of tumors.^9^, ^10^, ^11^, ^12^, ^13^ Accordingly, studies have shown that GSTA4 can promote the malignant progression of lung cancer, gastric cancer, colon cancer, and other tumors.^14^, ^15^ In liver cancer, for example, high expression of GSTA4 promotes tumor invasion and migration in vivo and in vitro by inducing Akt phosphorylation,^16^ but little is known about the role of GSTA4 in glioma.

In this study, we investigated the effect of curzerene on GSTA4 expression in glioma, and we explored antitumor effects and mechanisms of curzerene treatment in glioma cell lines and tumor‐bearing mouse models. This is the first report of the use of curzerene in glioma and also the first study on the relationship between the GSTA4 and malignant progression of glioma. This study will help in the identification of new drug candidates for the clinical treatment of glioma.

## 2 MATERIALS AND METHODS

### 2.1 Patient tissue sample collection

Clinical glioma samples were obtained from the Affiliated Hospital of Xuzhou Medical University. A total of 12 cases were confirmed by pathological diagnosis. Patients did not receive chemoradiotherapy before their operations. Clinical glioma samples were classified into low‐grade glioma (LGG) and glioblastoma (GBM) groups according to the 2016 World Health Organization Classification of Tumors of the Central Nervous System. Nontumor brain tissues from patients with acute brain injury undergoing intracranial decompression were obtained as a normal control group.

### 2.2 Cell culture and treatment

U251 and U87 cell lines were purchased from the cell bank of the Chinese Academy of Sciences, and human astrocyte (HA) cell lines were purchased from ScienCell Laboratory. U251 and U87 cells were cultured in high glucose Dulbecco's modified Eagle's medium (DMEM; Gibco) containing 10% fetal bovine serum (FBS; Gibco) and 1% penicillin and streptomycin (Beyotime) at 37°C and 5% CO_2_. HA cells were cultured in Ham's F‐10 nutrient mixture medium (Gibco) containing 10% FBS and 1% penicillin and streptomycin at 37°C and 5% CO_2_. For curzerene treatments, cells were treated with high glucose DMEM containing 50, 100, or 200 μM of curzerene for 12, 24, or 48 h.

### 2.3 RT‐PCR

Total RNA was extracted by the Trizol method (Invitrogen), and the RNA was reverse transcribed into cDNA with a Vic qRT Super Kit (Vicmed) in 20‐μl reactions. Real‐time quantitative PCR was performed with a LightCycler 480 (Roche), according to the manufacturer's instructions. Using GAPDH expression as an internal reference, the expression level of the target gene in each sample was calculated by the 2^−∆∆Ct^ calculation method. The sequences of primers were as follows: GSTA4 forward, 5′‐GTACAAGTTGCAGGATGGTAAC; GSTA4 reverse, 5′‐GAGATTGTGCTTGTCTGCTATG; GAPDH forward, 5′‐TGACTTCAACAGCGACACCCA; and GAPDH reverse, 5′‐CACCCTGTTGCTGTAGCCAAA.

### 2.4 Protein extraction and Western blotting

A total of 1 × 10^6^ cells were treated at 4°C for 30 min with 1 ml of lysis buffer containing protease and phosphatase inhibitors (Beyotime). The supernatant was collected by centrifugation at 24000g for 30 min. A BCA Kit (Beyotime) was used to quantify protein concentrations. Proteins were separated by gel electrophoresis and then transferred electrophoretically to a membrane. The membrane was blocked with blocking reagent (Beyotime) for 15 min, washed 3 times with washing buffer, and then incubated overnight with the primary antibody. The membrane was washed and then incubated with a fluorescently tagged secondary antibody for 2 h. Following a final wash, the membrane was scanned to determine the intensities of the bands. β‐Actin was used as an internal reference, and the intensity of the target protein was compared with the intensity of the internal reference in order to quantify the protein expression level.

### 2.5 Detection of cellular glutathione S‐transferase activity

Glutathione S‐transferase activity was quantified with a GST Activity Assay Kit (Solarbio) according to the manufacturer's instructions. Briefly, a total of 1 × 10^6^ cells were treated with 1 ml of activity detection reagent 1 and homogenized in an ice bath, and the precipitate was discarded. The supernatant (20 μl) was mixed with 180 μl of reagent 2 and 20 μl of reagent 3 in wells of a 96‐well plate. Activity was detected via increased absorbance at 340 nm in a plate reader.

### 2.6 Quantification of cellular 4‐hydroxynonenal

Cellular levels of 4‐hydroxynonenal (4‐HNE) were quantified with a detection kit (Jianglaibio) according to the manufacturer's instructions. Briefly, 1 × 10^6^ cells were resuspended in 1‐ml PBS, lysed with repeated freeze‐thaw cycles and centrifuged at 700g for 20 min. The enzyme‐coated test plate was prepared with 40‐μl diluent per well, and 10 μl of the clarified supernatant was added, followed by the addition of 100 μl of enzyme‐labeled reagent. The plate was sealed with sealing film and incubated at 37°C for 60 min. The sealing film was removed, the liquid was discarded, and each well was washed 5 times with detergent solution. Color reagent A (50 μl) was added to each well, followed by the addition of an equal volume of color reagent B. The reaction was mixed well with gentle shaking, and the color was allowed to develop at 37°C for 15 min. The reaction was terminated with 50 μl of stop solution. The absorbance (450 nm) of each well was determined, with a sample‐free well as a blank.

### 2.7 Assays of cell proliferation, migration, and invasion

Cell proliferation was detected by 5‐ethynyl‐2'‐deoxyuridine (EdU) fluorescence staining. Cells were inoculated in 96‐well plates at a density of 1 × 10^4^ per well and cultured overnight in a 5% CO_2_ incubator at 37°C. The next day, the cells were treated as noted. After treatment, the medium was replaced with EdU (Ribo) medium, and the cells were incubated for 2 h. After incubation, the cells were fixed for 30 min and washed. The cells were stained with EdU staining reagent Apollo^®^ (Ribo), and DAPI (Sigma Aldrich) was used to counterstain the nuclei. After staining, the percentage of EdU‐positive cells was counted.

The migration ability of cells was detected by a wound healing test. Cells were seeded at a density of 1 × 10^5^ per well on a 6‐well plate, and a scratch was induced with a pipette gun. After culturing in serum‐free medium for 0 and 24 h, the wound healing area was observed under a microscope.

A transwell migration assay was used to detect cell invasion. Matrix glue (Corning) was diluted in an equivalent volume of serum‐free medium, and 50 μl of the diluted matrix glue was incubated in the upper chamber of a transwell apparatus for 2 to 3 h until the gel solidified. Cells were digested and counted, and a cell suspension was prepared with serum‐free medium. Complete medium containing 20% FBS was added to the lower chamber. A cell suspension (200 μl) was added to the upper chamber of each well, and the medium was changed to drug‐containing medium after the cells adhered to the well. The cells were cultured at 37°C for 24 h and then fixed for 10 min. Cells were stained with 0.1% crystal violet for 30 min at room temperature, and cells on the upper surface were removed by wiping with a cotton ball. Cells were observed, photographed, and counted with an inverted microscope.

### 2.8 Cell inhibition and detection of apoptosis

Cells were seeded into 96‐well plates at a density of 1 × 10^4^ cells per well and were treated with drugs the following day. After treatment for the noted time, 20 μl of CCK8 reagent (Vicmed) was added to each well, the plate was incubated in the dark for 30 min, and the absorbance at 450 nm was detected by a microplate reader.

Apoptosis was measured via a TUNEL kit (KeyGen), according to the manufacturer's instructions. After treatment, the cells were fixed with 4% paraformaldehyde (KeyGen) for 30 min, and the cells were then permeabilized for 5 min. TDT enzyme reaction solution (KeyGen) was added to each well, and reactions were incubated in the dark at 37°C for 1 h. The streptavidin TRITC labeling solution (KeyGen) was added, and the mixture was incubated in the dark in a humidified environment at 37°C for 30 min. After washing three times with PBS, DAPI was added for 10 min to counterstain nuclei. The results were observed with a fluorescence microscope.

### 2.9 Quantification of cellular GSH

GSH （Glutathione） was quantified using a detection kit (Jiancheng) according to the manufacturer's instructions. Briefly, treated cells were collected, and the cell pellet volume was estimated visually. Phosphate‐buffered saline (PBS; 4 volumes) was added to resuspend the cells, and cells were disrupted with an ultrasonic cell crusher. The cell lysate and detection reagents were added to wells of a 96‐well plate according to the manufacturer's instructions, the contents were mixed, and the absorbance at 405 nm was measured with a microplate reader.

### 2.10 In vivo test

The Institutional Animal Care and Use Committee of the Xuzhou Medical University approved all experimental protocols (approval No. 202009A164) on September 16, 2020. All experimental procedures were in accordance with the guidelines for laboratory animal welfare and use set by the Ministry of Health of China and the relevant guidelines of the National Institutes of Health. All reporting of animal data followed the Animal Research: Reporting of in vivo experiments guidelines.^17^

Male BALB/C nude mice (aged 6 weeks, weighing 18–22 g) were purchased from Beijing Weitong Lihua Experimental Animal Technology Co., Ltd. All BALB/C nude mice were caged in a pathogen‐free animal laboratory. The health status of nude mice was observed prior to each experiment. During the feeding process, nude mice were randomly divided into two groups. Curzerene was diluted to a concentration of 1 mM with saline. A suspension of 1 × 10^6^ U251 cells was injected into the armpits of mice. Two weeks after tumor formation, the mice were injected intraperitoneally every other day with curzerene diluted in normal saline at doses of 0.1 ml per 10 g bodyweight. Tumor size and weight were measured twice per week for 4 weeks. The tumor volume was calculated according to the following formula: (length) × (width)^2^/2. After 4 weeks, the mice were sacrificed and the tumor was isolated.

### 2.11 Immunohistochemical staining

Formalin‐fixed and paraffin‐embedded subcutaneous tumor sections were dewaxed, rehydrated, and stained and boiled in 10‐mM sodium citrate buffer (pH 6.0) for antigen recovery. The sections were stained with GSTA4, Ki67, p‐mTOR, MMP9, and Bcl‐2 antibodies and then incubated with a biotin secondary antibody. The slides were dipped into solutions containing horseradish peroxidase and 3, 3′‐diaminobenzidine (DAB) successively, stained with hematoxylin, dehydrated, and sealed with neutral gum. The results were observed under a microscope.

### 2.12 Statistical analysis

Data were analyzed with SPSS 22.0 or GraphPad Prism 8 software and expressed as mean ± standard deviation. Data normality was tested using the Shapiro‐Wilcoxon normality test, rejecting the normality at *p* < 0.05. The Student's *t*‐test and one‐way ANOVA test were used to assess the significance of differences between groups. Kaplan‐Meier survival analysis was used to estimate the survival distributions, and the log‐rank test was used to assess the statistical significance between stratified survival groups. In all statistical analyses, a two‐sided *p* value <0.05 was considered to indicate statistical significance.

## 3 RESULT

### 3.1 GSTA4 is highly expressed in gliomas, and expression level correlates with tumor grade

**FIGURE 1:**
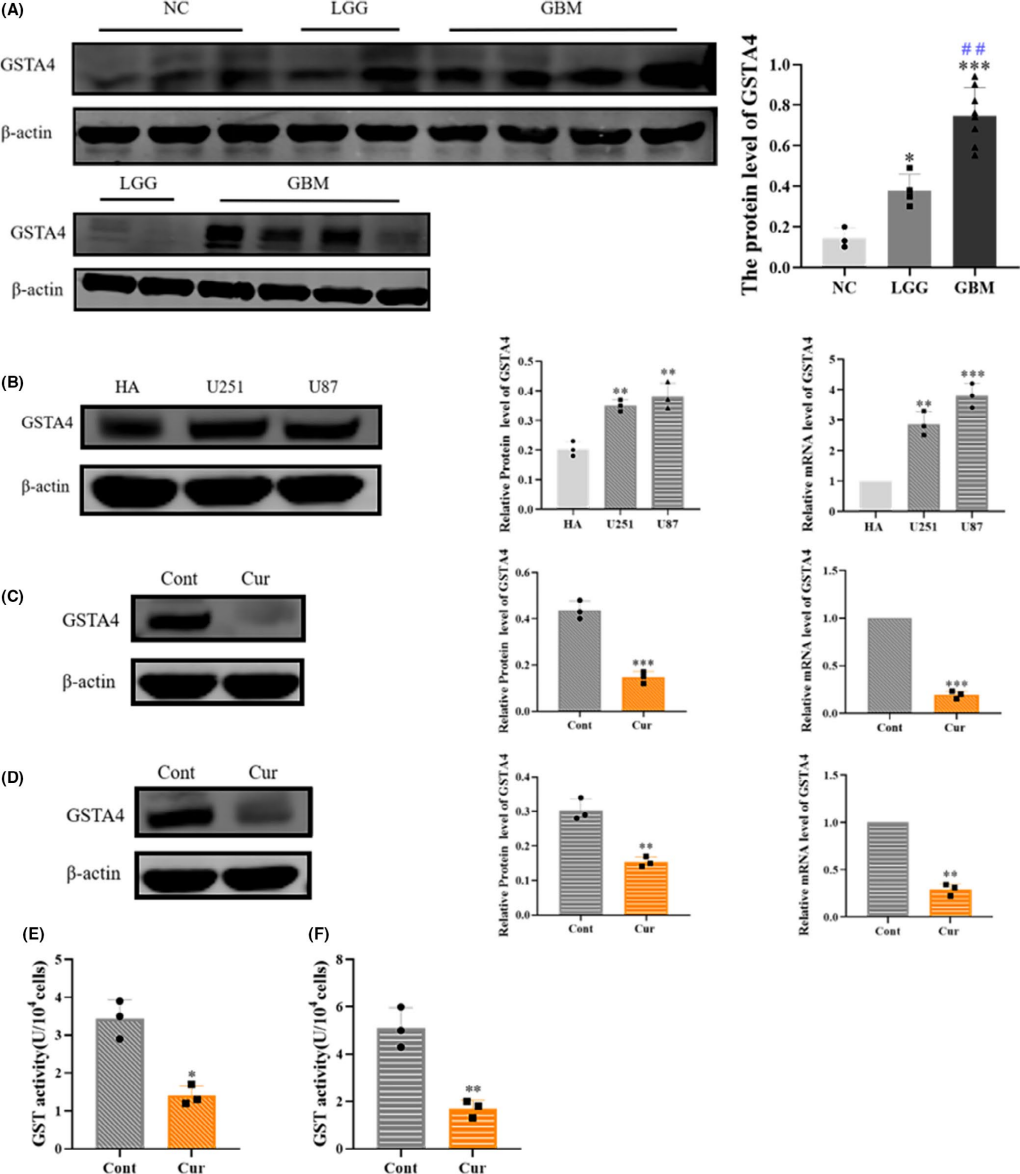
Glutathione S‐transferase A4 (GSTA4) is highly expressed in gliomas, and expression is inhibited by curzerene. (A) The expression level of GSTA4 in low‐grade glioma was lower than that in glioblastoma (LGG vs. GBM, # #: *p* < 0.01). (B) GSTA4 was highly expressed in U251 and U87 cells. (C and D) Curzerene inhibited the expression of GSTA4 in U251 and U87 cells. (E and F) Curzerene inhibited the GST activity of in U251 and U87 cells. *p < 0.05; **p < 0.01; ***p < 0.001

GSTA4 is abnormally expressed in and affects the progression of a variety of malignant tumors. In order to confirm whether the expression of GSTA4 is upregulated in malignant gliomas, we detected the expression of GSTA4 in four cases of low‐grade gliomas and eight cases of glioblastomas by Western blot. The results showed that the expression level of GSTA4 in tumor tissue was significantly higher than that in normal brain tissue. Further comparison of the expression levels of GSTA4 in low‐grade gliomas and glioblastomas showed that the expression level of GSTA4 in glioblastomas was significantly higher than that in low‐grade gliomas (Figure 1A).

### 3.2 Curzerene inhibits the expression and activity of GSTA4 in glioma cell lines

In order to determine whether curzerene affects the expression of GSTA4 in glioma, we selected the U87 and U251 glioma cell lines as experimental subjects. First, Western blot and RT‐PCR analyses were used to detect the expression of GSTA4 in U87, U251, and nonglioma HA cells. The results showed that GSTA4 was more highly expressed in U87 and U251 cells as compared to HA cells. After treatment with curzerene, the expression of GSTA4 protein in U251 and U87 cells was significantly inhibited (Figure 1B‐D). Further detection of cellular GST enzyme activity showed that curzerene treatment can significantly downregulate the overall GSH‐metabolizing catalytic ability of the two glioma cell lines (Figure 1E,F).

### 3.3 Curzerene downregulates the antioxidant function of GSTA4 and induces apoptosis

GSTA4 can protect cells from oxidative stress products and can detoxify 4‐HNE in normal cells and many cancer cells by catalyzing the formation of an adduct of GSH and 4‐HNE. We hypothesize that curzerene, a known GSTA inhibitor, can reduce the linking of GSH and 4‐HNE by downregulating the expression of GSTA4 and lowering the enzyme activity in cells. This downregulation would result in increased concentrations of free 4‐HNE and the promotion of 4‐HNE‐induced apoptosis of tumor cells.

**FIGURE 2:**
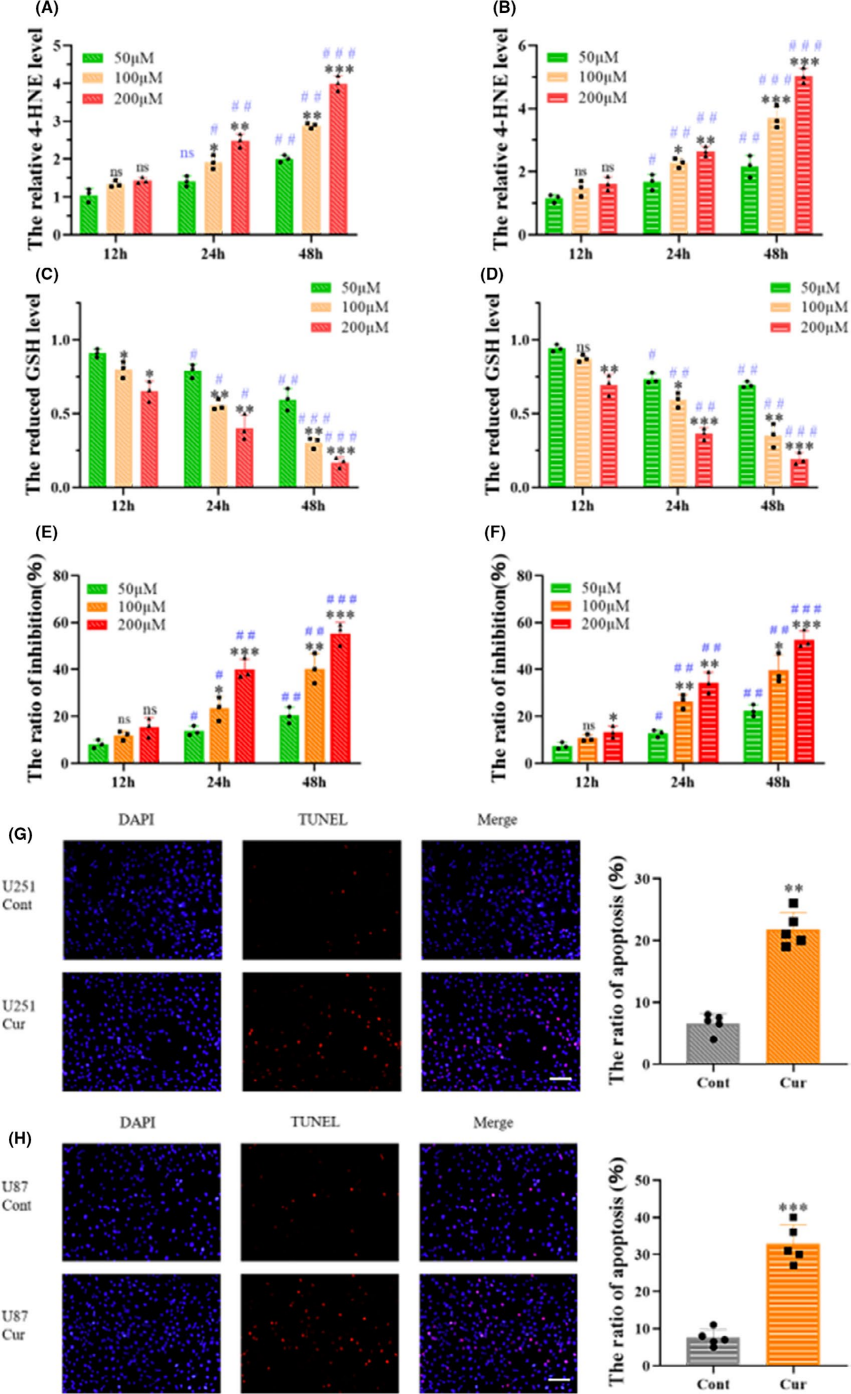
Curzerene inhibits antioxidant functions in glioma cells and induces apoptosis. (A‐D) Curzerene treatment increased intracellular levels of 4‐HNE and reduced the level of GSH in a time‐ and dosedependent manner (12 h vs. 24 h, 12 h vs. 48 h, #*p* < 0.05, # #: *p* < 0.01, # # #: *p* < 0.001). (E and F) CCK‐8 results showed that curzerene inhibited the viability of U251 and U87 cells in a time‐ and dose‐dependent manner (12 h vs. 24 h, 12 h vs. 48 h, # *p* < 0.05, # #: *p* < 0.01, # # #: *p* < 0.001). (G and H) TUNEL assays showed that curzerene correlated with increased apoptosis in U251 and U87 cells. *p < 0.05; **p < 0.01; ***p < 0.001

Consistent with this hypothesis, the results of a 4‐HNE assay showed that the level of 4‐HNE in both glioma cell lines increased after curzerene treatment (Figure 2A,B). Interestingly, although the amount of GSH bound to cellular 4‐HNE was reduced, the effect of curzerene also reduced the level of free GSH in cells (Figure 2C,D). This effect may be induced by inhibition of GSTA4 expression, or it may be that the peroxide produced following curzerene treatment consumes GSH through glutathione peroxidase activity or other pathways.^18^

We used a CCK‐8 assay to investigate curzerene's inhibitory effect on cell viability, and our results demonstrate that curzerene treatment inhibits the viability of U251 cells in a dose‐ and time‐dependent manner. The difference in viability was significant at both 24 and 48 h, and the cell inhibition rate was highest upon treatment with 200 μM of curzerene. In U87 cells, the concentration of curzerene and time of treatment were also positively correlated with the cell inhibition rate. In these cells, the difference in viability was statistically significant at 48‐h treatment time (Figure 2E,F). TUNEL staining showed that the apoptosis levels of U251 and U87 cells were significantly increased after curzerene treatment for 24 h. The apoptosis rate of U251 cells increased from 5% to 24% and that of U87 cells increased more than 3 fold (Figure 2G, H).

### 3.4 Curzerene reduces the proliferation, migration, and invasion of glioma

**FIGURE 3:**
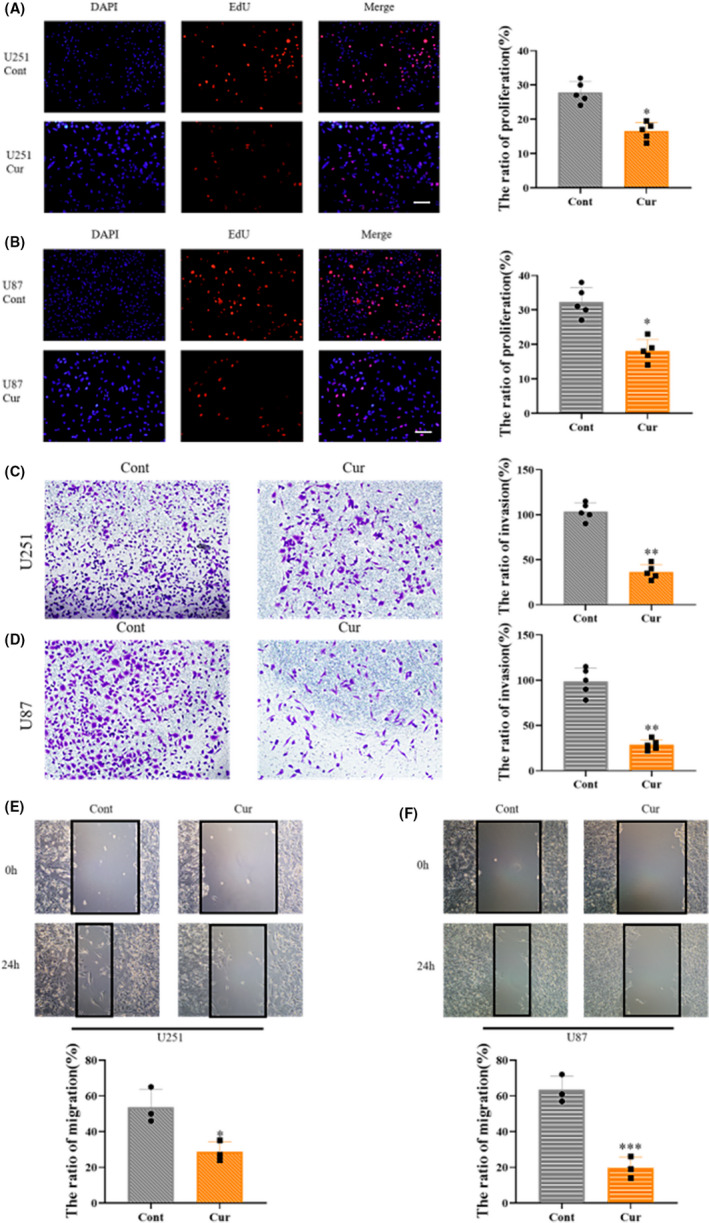
Curzerene inhibits the proliferation, invasion and migration of U251 and U87 cells. (A and B) EdU assays demonstrated inhibition of the proliferation of U251 and U87 cells by curzerene treatment. (C and D) In transwell migration assays, the ability of U251 and U87 cells in the curzerene treatment group to cross matrix gel was lower than that of the control group. (E and F) Wound healing experiments showed that curzerene treatment lowered the migration ability of U251 and U87 cells. *p < 0.05; **p < 0.01; ***p < 0.001

Malignant gliomas have a strong ability to proliferate, invade, and migrate. We, therefore, investigated the effect of curzerene treatment on these malignant characteristics of glioma. EdU staining was used to evaluate cell proliferation. The results showed that the proliferation of U251 cells decreased from 27% to 16% after treatment with curzerene for 24 h. Curzerene treatment also reduced proliferation of U87 by approximately 30% (Figure 3A,B). Wound healing assays showed that the migration abilities of U251 and U87 cells were reduced by 42% and 34%, respectively, after curzerene treatment (Figure 3C,D). In transwell migration assays, curzerene treatment reduced the number of cells passing through the matrix gel. This latter effect was observed in both U87 and U251 cell line (Figure 3E,F).

### 3.5 Curzerene inhibits the activation of the mTOR pathway and the expression of MMP9

**FIGURE 4:**
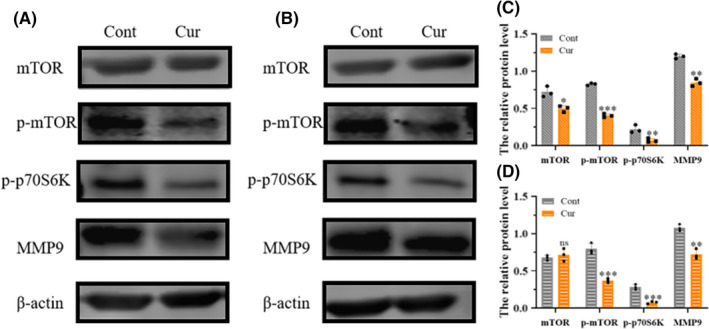
Curzerene inhibits the mTOR pathway and MMP9 expression. (A‐C) The effect of curzerene on the activation of mTOR and the expression of MMP9 in U251 cells was detected by Western blotting. (B, D) The effect of curzerene on the activation of mTOR and the expression of MMP9 in U87 cells was detected by Western blotting. *p < 0.05; **p < 0.01; ***p < 0.001

In order to understand the pathway by which curzerene inhibits cell proliferation, we used Western blotting to detect the levels of activation of molecules in the mTOR pathway in the glioma cell lines. The results showed that curzerene treatment only slightly inhibited the level of total mTOR in U251 and U87 cells, but it significantly reduced the phosphorylation of mTOR in both cell lines. Meanwhile, the phosphorylation of p70S6 kinase, a downstream factor of mTOR, was also significantly decreased, indicating that the activation of the mTOR/p70S6K axis was inhibited. Using Western blot assays, we also found that curzerene inhibited the expression of matrix metalloproteinase 9 (MMP9) in U251 and U87 cells (Figure 4A‐D).

### 3.6 Curzerene downregulates glioma proliferation by inhibiting the mTOR pathway

**FIGURE 5:**
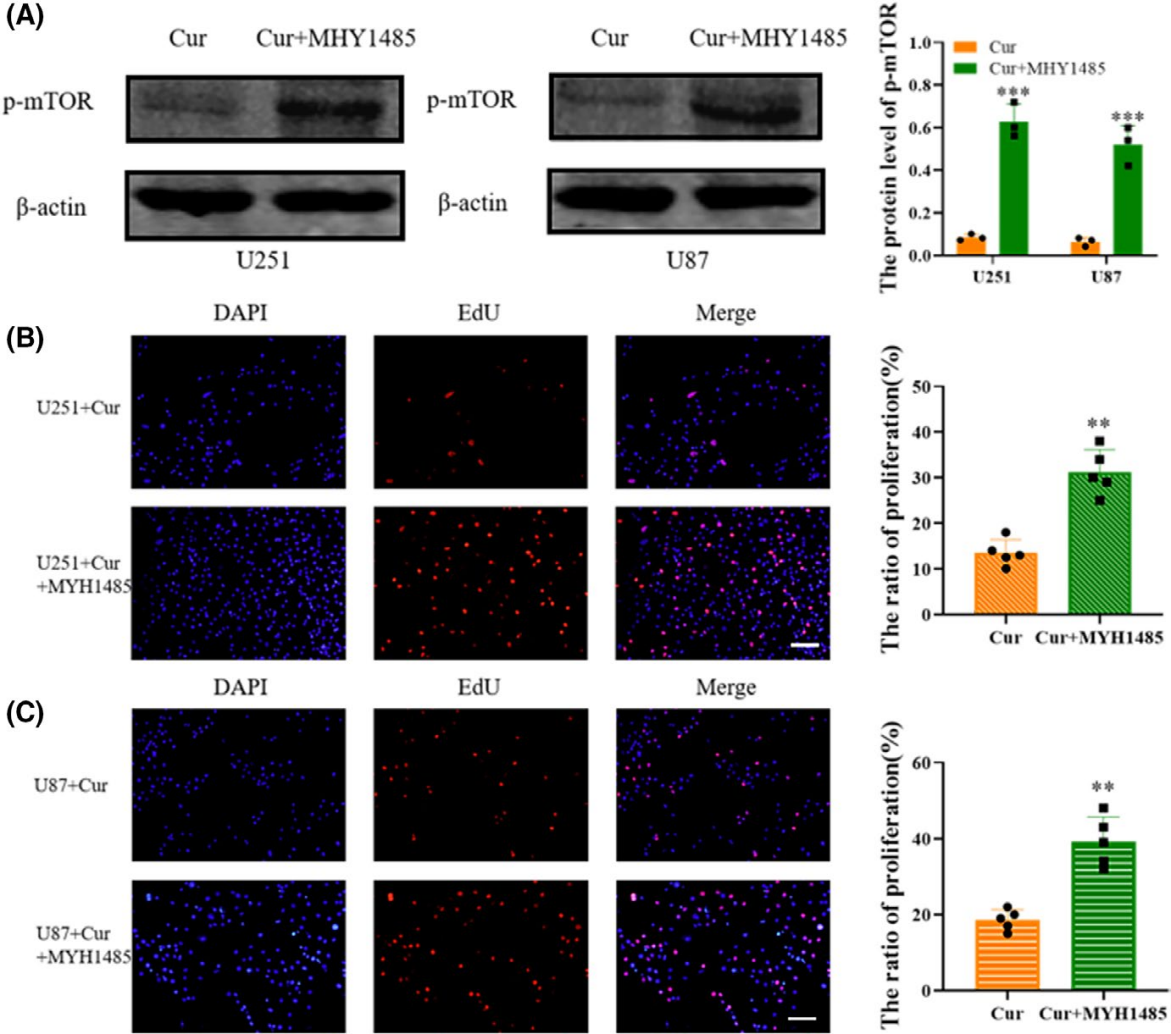
Curzerene inhibits mTOR phosphorylation and down regulates glioma proliferation. (A) Western blotting was used to detect the effect of an mTOR agonist on the level of p‐mTOR in U251 and U87 cells. (B and C) The effect of the mTOR agonist on curzerene‐induced cell proliferation inhibition was detected by EdU assays. **p < 0.01; ***p < 0.001

In order to determine whether the inhibition of cell proliferation is induced through curzerene‐mediated inhibition of mTOR phosphorylation, we treated U251 and U87 cells with mTOR agonist MHY1485. The effectiveness of MHY1485 in increasing phosphorylation of mTOR was verified by Western blotting (Figure 5A). Subsequent EdU results demonstrated that the inhibition of U251 and U87 cells by curzerene was reversed after the activation of mTOR. These results are consistent with a model in which curzerene‐induced inhibition of proliferation was caused by the downregulation of phosphorylation and inactivation of the mTOR/p70S6k axis (Figure 5B,C).

### 3.7 Curzerene inhibits the growth of transplanted tumors in nude mice

**FIGURE 6:**
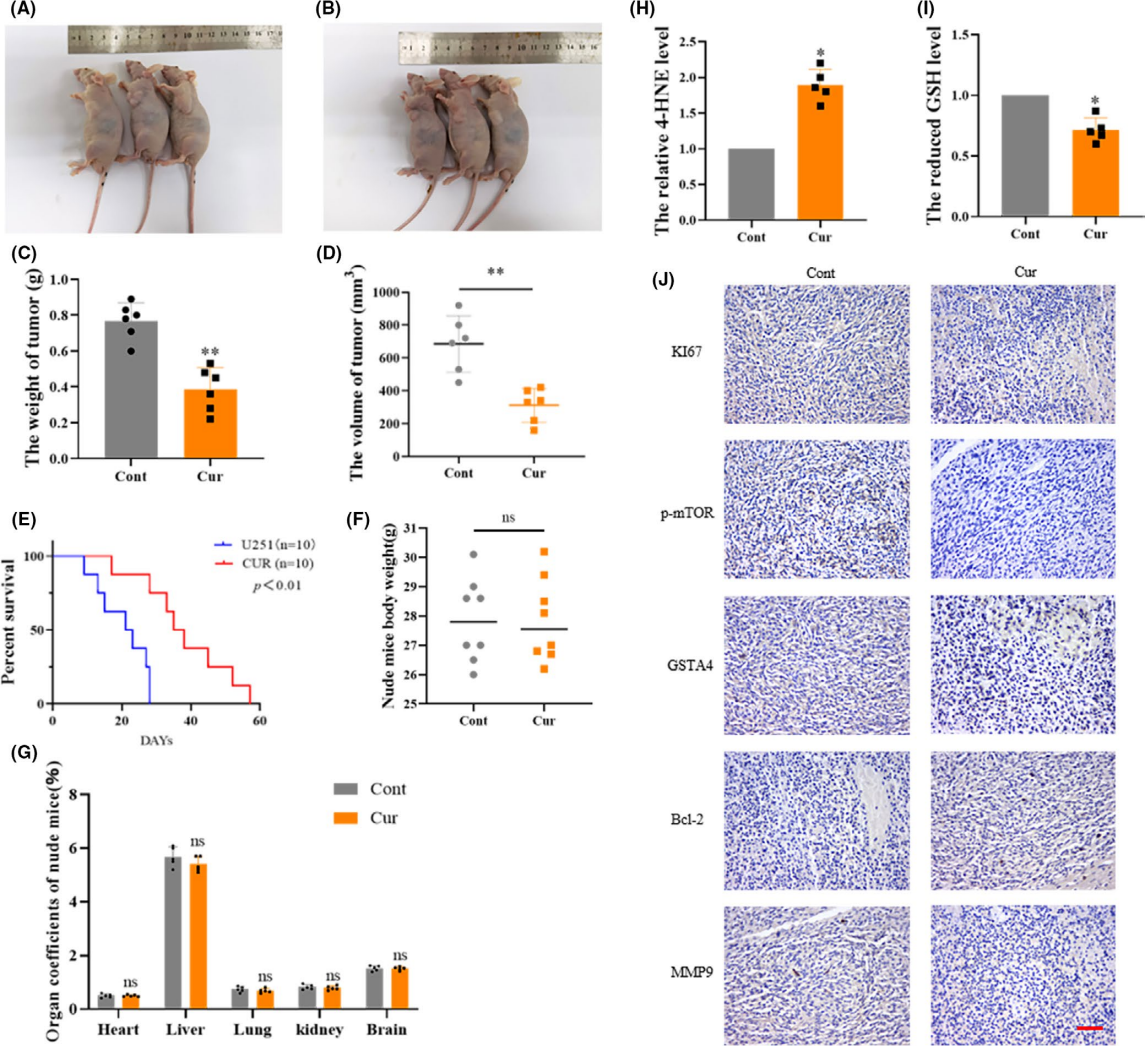
The antitumor effect of curzerene in a nude mouse tumor model. (A and B) Mice from the control group (A) and curzerene treated group (B) were sacrificed 4 weeks after tumor formation. (C‐E) The effects of curzerene on the weight and volume of subcutaneous transplanted tumors and the survival time of tumor bearing nude mice were compared statistically. (F and G) The effects of curzerene on the body weights and weights of internal organs of curzerene‐treated and control mice were compared. (H and I) The effects of curzerene on the 4‐HNE level and GSH level were compared. (J) Immunohistochemistry was used to detect the effect of curzerene on various molecules in tumors of nude mice. ns *p* > 0.05, *p < 0.05; **p < 0.01

In order to test the potential in vivo tumor inhibitory effect of curzerene, we injected U251 cells into the flanks of nude mice to establish a subcutaneous xenograft tumor model. The nude mice were randomly divided into two groups. After 2 weeks of tumor growth, normal saline or a 1 mM curzerene solution was injected intraperitoneally once every 2 days. By observing the tumor volume and survival time of the tumor‐bearing nude mice, it was found that the tumor volume and weight in the curzerene treatment group were significantly lower than those of the normal saline control group (Figure 6A‐D). Meanwhile, injection with curzerene significantly prolonged the survival time of tumor‐bearing mice (Figure 6E). In order to detect the drug toxicity of curzerene at a therapeutic dose in nude mice, nude mice were injected intraperitoneally once every 2 days for 2 weeks with curzerene or physiological saline. The mice were sacrificed after the treatment, and body weights and weights of internal organs were analyzed and were found to not be significantly different between curzerene and control groups (Figure 6F,G).

Tumor samples from nude mice were analyzed by immunohistochemistry. The expression of GSTA4, Ki67, and MMP9 and the levels of p‐mTOR were significantly decreased in nude mice treated with curzerene relative to the control mice, while Bcl‐2 expression was significantly increased (Figure 6J). Curzerene also decreased the GSH levels and increased 4‐HNE levels in xenografts (Figure 6H,I). These results suggest that in addition to downregulating the proliferation, invasion, and migration of glioma cell lines in vitro, treatment with the GSTA4 inhibitor curzerene also leads to mitigation of tumor growth, invasion, and viability in vivo.

## 4 DISCUSSION

Our results show that GSTA4 is highly expressed in gliomas and that its expression level is positively correlated with the degree of malignancy of gliomas. Curzerene can induce apoptosis by inhibiting the expression of GSTA4 in gliomas, and it can downregulate the proliferation, invasion, and migration of gliomas by inhibiting the phosphorylation and activation of mTOR and the expression of MMP9.

Curzerene is a sesquiterpenoid that was originally extracted from the traditional Chinese medicine longan. Studies have shown that curzerene treatment can reduce the secretion of tumor necrosis factor in THP‐1 cells, suggesting that it has potential as an anti‐inflammatory therapy.^8^ In addition, low concentrations of curzerene may inhibit the release of nitric oxide from macrophages and then affect the activation of cell signaling.^8^, ^19^ Most recently, curzerene has been shown to inhibit the progression of non‐small‐cell lung cancer.^8^ These multiple positive results suggest that curzerene is a safe and effective therapeutic agent, which makes its potential application to a disease as deadly as glioma very interesting.

The results here show that GSTA4 is highly expressed in both low‐grade gliomas and glioblastomas, but that the expression level of GSTA4 in glioblastomas is much higher than that in low‐grade gliomas. This positive correlation of expression with degree of malignancy of gliomas suggests that GSTA4 can promote the occurrence and development of gliomas. High levels of GSTA4, thus, may predict poor prognosis of disease progression in glioma patients. Notably, GSTA4 can induce lens epithelial carcinogenesis, and it is also a risk factor for nonmelanoma skin cancer.^20^ Similarly, it is highly expressed in lung cancer, gastric cancer, breast cancer, and cervical cancer.^21^

Curzerene has been shown to inhibit GSTA expression in multiple cell types, including lung cancer.^8^ Specifically with regard to lung cancer, the downregulation of the expression of *GSTA1* by curzerene correlates with an inhibition of tumor proliferation and migration.^8^ In this study, we found that curzerene can significantly inhibit the expression of GSTA4 in the glioma cell types U251 and U87. GSTA4 is an important biphasic detoxification enzyme in human body, which is involved in the combination of GSH with reactive oxygen species, cell metabolites, chemotherapy drugs, and other potentially damaging molecules.^22^, ^23^, ^24^, ^25^, ^26^, ^27^, ^28^, ^29^

Recently, GSTA4 has also been shown to affect the progression of colon cancer, as well, and this effect involves the proapoptotic metabolite 4‐HNE. Specifically, in colon cancer, activator protein 1 regulates the production of 4‐HNE by affecting the c‐Jun/Nrf2 complex, which then results in an increase in the level of GSTA4 in cells.^9^ In the presence of increased GSTA4 activity, then 4‐HNE is more rapidly degraded, and apoptosis is inhibited. Our study found that curzerene‐induced inhibition of GSTA4 expression can significantly induce tumor apoptosis by upregulating the level of 4‐HNE and reducing the level of reduced GSH, indicating that the high expression of GSTA4 enhances the ability of glioma to resist oxidative stress injury and that GSTA4 plays an important role in the maintenance of tumor survival.

In a variety of tumors, such as drug‐resistant cervical cancer, the expression of GSTA4 is increased, suggesting that GSTA4 is related to tumor survival and drug resistance.^30^, ^31^ Our study reached a similar conclusion in that inhibition of GSTA4 expression by curzerene‐inhibited cell activity and upregulated apoptosis. GSH is a key reducing agent, and GSTA4 plays a detoxification role by catalyzing the reduction of toxic molecules using GSH as source of reducing equivalents. High levels of GSH promotes the malignant progression and drug resistance of a variety of tumors, including glioma.^32^, ^33^ Contrary to the expected results, our study showed that curzerene treatment can reduce the level of intracellular reduced GSH, which may be due to the upregulation of other intracellular oxidative stress products, such as H_2_O_2_, the consumption of GSH by the glutathione peroxidase pathway, or the direct inhibition of intracellular GSH by downregulation of GSTA4. These results further suggest that curzerene may regulate the apoptosis of glioma by changing the redox level of cells.^34^, ^35^, ^36^ At the same time, the combination of curzerene and chemotherapy drugs may improve the drug sensitivity of tumor and promote the sensitivity of drug‐resistant glioma to chemotherapy drugs.^37^, ^38^

In a recent study, Liu et al.^16^ found that the high expression of GSTA4 promoted the malignant progression of liver cancer and promoted the invasion and migration of the tumor through phosphorylation and activation of Akt. Interestingly, our results show that curzerene induces decreased phosphorylation of mTOR in gliomas and inhibits its downstream signal transduction. The mTOR pathway is an important carrier of signals leading to cell proliferation, and it is highly activated in a variety of tumors and promotes the malignant progression of tumors.^39^ The inhibitory effect of curzerene on cell proliferation was reversed upon treatment with an mTOR agonist, indicating that curzerene's ability to inhibit glioma proliferation depends on the downregulation of the mTOR/p70S6K axis. It is worth noting that there has been no research that has connected the activation level of mTOR and the expression of GSTA4; as an inhibitor of GSTA4, therefore, whether curzerene can inhibit mTOR by downregulating GSTA4 remains to be further explored.

Our study also found that curzerene downregulated the expression of MMP9 in glioma. MMP9 is widely expressed in various malignant tumors, and it promotes tumor invasion and migration by degrading the extracellular matrix. Studies have shown that the high invasion and migration ability of glioma is related to the high expression of MMP9.^40^, ^41^, ^42^ The downregulation of MMP9 in cell lines and tumor tissues may explain the mechanism by which curzerene inhibits glioma invasion and migration. Studies have shown that the expression of GST family enzymes and MMP9 are early events in the development of esophageal cancer.^43^, ^44^ Induction of prostate cancer also correlates with increased levels of GSTP and MMP9.^45^ It is therefore worth exploring whether there is a similar relationship between GSTA4 and MMP9 in glioma.

There were several limitations to this study. Although the commercial GBM cell line used in the mouse subcutaneous tumorigenesis model and in vitro studies is widely recognized as an appropriate research model for GBM, there are significant differences in molecular heterogeneity and chemosensitivity between these cells and clinical GBM.^46^ Notably, however, some newly discovered molecular markers have been proven to be of great value in the study of prognostic correlations in GBM, including TRPM7, CXCL1, and COPB2.^47^, ^48^, ^49^, ^50^, ^51^ In follow‐up studies, we will use additional primary GBM cells as models, and we will detect the chemoresistance and prognosis of clinical GBM through comprehensive analysis of prognosis‐related molecular markers and noninvasive imaging‐based biomarkers such as MRI detection of oxygen metabolism and neovascularization.^52^, ^53^

The results of our in vivo experiments were consistent. Curzerene‐treated tumor tissues showed decreased GSTA4 levels and decreased proliferation, invasion, and migration, accompanied by increased apoptosis, decreased GST catalytic activity, and increased 4‐HNE. These factors correlated with a prolonged survival time of tumor‐bearing nude mice, which had significantly inhibited tumor growth. Importantly, this treatment did not significantly reduce the body weight or organ weight of nude mice. These results indicate that curzerene, as a traditional Chinese medicine extract, can inhibit the progression of glioma.

## 5 CONCLUSION

Curzerene, as a traditional Chinese medicine extract, can inhibit the malignant progression of glioma by downregulating GSTA4, p‐mTOR, MMP9, and other signals. Curzerene is a promising candidate for a new drug to treat glioma.

## CONFLICTS OF INTERESTS

The authors declare no conflicts of interest.

## AUTHOR CONTRIBUTIONS

Bo Cheng is the investigative lead responsible for the research ideas, design, manuscript write up, and experimental works, Xiaoliang Hong, Linfang Wang, Yuanyuan Cao, and Dengli Qin carried out major research experiments, and analysis as well as results arrangement under the supervision and guidance of Dianshuai Gao. Other co‐author helped in data gathering and analysis. All authors have read and approved the final manuscript.

## Data Availability

The data that support the findings of this study are available from the corresponding author upon reasonable request.
